# Pediatric Oncology Palliative Care: Experiences of General Practitioners and Bereaved Parents

**DOI:** 10.4172/2165-7386.1000214

**Published:** 2015-03-01

**Authors:** Sue J. Neilson, Faith Gibson, Sheila M Greenfield

**Affiliations:** 1 Birmingham Children’s Hospital NHS Foundation Trust, Steelhouse Lane, Birmingham, B4 6NH, UK; 2 Centre for Outcomes and Experience Research in Children’s Health, Illness and Disability, Great Ormond Street Hospital for Children NHS Foundation Trust, Faculty of Health and Social Care, London South Bank University, 103 Borough Road, SE1 0AA, London; 3 School of Health and Population Sciences, University of Birmingham, Edgbaston, Birmingham, B15 2TT, UK

**Keywords:** Children, Palliative care, Cancer, Oncology, General practitioners, Qualitative

## Abstract

**Objective:**

This qualitative study set in the West Midlands region of the United Kingdom, aimed to examine the role of the general practitioner (GP) in children’s oncology palliative care from the perspective of GPs who had cared for a child with cancer receiving palliative care at home and bereaved parents.

**Methods:**

One-to-one semi-structured interviews were undertaken with 18 GPs and 11 bereaved parents following the death. A grounded theory data analysis was undertaken; identifying generated themes through chronological comparative data analysis.

**Results:**

Similarity in GP and parent viewpoints was found, the GPs role seen as one of providing medication and support. Time pressures GPs faced influenced their level of engagement with the family during palliative and bereavement care and their ability to address their identified learning deficits. Lack of familiarity with the family, coupled with an acknowledgment that it was a rare and could be a frightening experience, also influenced their level of interaction. There was no consistency in GP practice nor evidence of practice being guided by local or national policies. Parents lack of clarity of their GPs role resulted in missed opportunities for support.

**Conclusions:**

Time pressures influence GP working practices. Enhanced communication and collaboration between the GP and regional childhood cancer centre may help address identified GP challenges, such as learning deficits, and promote more time-efficient working practices through role clarity. Parents need greater awareness of their GP’s wide-ranging role; one that transcends palliative care incorporating bereavement support and on-going medical care for family members

## Background

Providing palliative care to adults is seen as an important component of general practitioners’ (GP’s) practice as, although palliative medicine is recognized as an established specialty, it is unlikely that all patients requiring palliative care will be managed by a specialist [1]. “Palliative care” is used here to describe the holistic multi-professional care given to children and their families, and “palliative medicine” the medical specialty of doctors working solely with patients with life-limiting conditions. Pediatric palliative medicine (PPM) is less well established than adult palliative medicine (only being recognized as a subspecialty for doctors in 2009 [2]) and providing palliative care to children often poses challenges to GPs [3]. The challenges can be attributed in part to the infrequency of child deaths in the community but may also be influenced by the lack of pediatric training UK GPs undertake (only 40-50% of GPs complete a pediatric placement during training [4]). Childhood cancers, although rare, cause the largest numbers of deaths by disease in children aged 1-19 years [5] with more than 400 children a year dying from cancer in the UK [6]. The incidence of childhood cancer deaths and infancy of PPM as a specialty, coupled with parental preference for their child to die within the family home [6] highlight the importance of the GPs role in this highly specialized area of clinical practice.

Although nationally in the UK there is a recognized lack of standardization in children’s palliative care provision [7] exemplars of specialist teams for defined disease groups exist, such as within children’s cancer care [6]. Here there is a defined model for providing palliative care where pediatric oncology outreach nurse specialists (POONS) take prominence in coordinating care [6]. POONS work between primary, secondary and tertiary care providing specialist advice (such as symptom management) and support to families and health professionals. The GP however has a unique role as the only health professional that has long-term involvement with the family; their role transcending palliative care incorporating bereavement support and on-going medical care for family members.

The rarity of childhood cancer, coupled with the child’s first port of call during cancer treatment being the hospital, can result in GPs feeling excluded during the treatment period, despite the recognition of their role in supporting the whole family [3,8]. Although GPs themselves recognize they have a role to play and can be of value in providing palliative care [3,8] it has been shown that their ability to fulfill this role can be hindered by a lack of anticipation of involvement and negotiation of roles and responsibilities at the child’s transition to palliation (when interventions are no longer aimed at cure) and knowledge deficits [3].

GP practices with an interest in palliative care are most likely to have comprehensive, proactive bereavement policies and both identify and routinely contact the bereaved [9]. Active involvement in palliative care can aid GPs to initiate bereavement support [10], their role encompassing identifying abnormal grief reactions and providing preventative care [9,10].

Studies examining parents’ views of their child’s end of life highlight the importance of effective symptom management, collaborative inter-professional working and communication [11-14]. Little is known about parents’ views of the GP’s role in children’s cancer palliative and bereavement care yet their experience could inform GP practice and PPM. Implementation of the as yet unmet recommendation for regional consultants and locality pediatricians with specialist interests in this field [15] could be beneficial to GPs in the future. Understanding bereaved parents’ perceptions provides a unique perspective; the value of listening to user groups and associated benefits to informing practice and developing service delivery being well recognized [16,17].

Here we present the views of the GPs and parents and suggest how findings could inform not only GP practice, but the future development of PPM.

## Methods

This study was approved by South Birmingham Research Ethics Committee (10/H1207/25) and by the Research and Development departments of the recruitment sites.

### Study design

This qualitative study used semi-structured interviews and ground theory analysis to explore the experiences of GPs and parents following the death of a child from cancer within the family home. Using grounded theory informed both the sampling and data analysis, through providing a systematic research approach for collecting and analyzing the qualitative data [18]. Adopting this approach ensured that what was relevant to the study arena was allowed to emerge rather than focusing on setting out to prove a defined theory [19].

### Setting

The geographical setting was determined by referrals to a UK regional childhood cancer Centre (RCCC).

### Sample

Within grounded theory, sampling aims to seek anticipated potential variation in the range of experiences within the practical constraints of available cases, thereby in this study aiming for a representative subsection of range of diagnoses and GP characteristics.

The sample comprised bereaved parents whose child (aged 0-18 years) had been treated at a UK RCCC and died within the family home, and the associated GP. Data were collected over fifteen months; the sample selected chronologically following the child’s death. Families on the clinical caseload held by the researcher (SN), POONS, were excluded from the sample.

### Data collection

The researcher was informed of the child’s death through the POONS team at the RCCC. Information sheets were posted to the GPs three, and the parents six months after the child’s death and followed by a telephone call one week later. The parent information sheet was revised after three parents declined participation. The shortened letter briefly introduced the researcher, outlined the study and detailed means of declining further contact. The information sheets explained that the respective parent/GP was also being invited to participate but no further details provided. The date, time and venue for the interview were confirmed with those agreeing to participate.

Informed written consent was obtained prior to commencing the tape-recorded semi-structured interviews. Separate interview schedules were used for GPs and parents. Both started with an opening question asking them to describe their involvement in the care (GPs) or the care that their child received at home (parents). Follow-up questions were asked in order to gain clarification of a particular point or to explore a new topic direction. A topic guide provided additional questions such as, questions on the perceived/actual GP role in the care (Table 1). Interview duration ranged from 40-120 minutes, with the majority of GP interviews lasting less than one hour. Afterwards GPs completed a demographic data questionnaire and parents were given a bereavement support leaflet. All participants were offered a support telephone call one week later.

### Data analysis

The tapes were professionally transcribed verbatim and then checked to ensure accuracy. Using NVivo data were analyzed concurrently; the transcripts examined line-by-line, sentence-by-sentence and paragraph-by-paragraph. Data were initially placed under broad headed categories (codes), which gathered together all the relevant information from the interview to that topic. Following a grounded theory approach [20,21] data were compared and contrasted with each new transcript. Throughout this process the codes evolved and concepts were linked, until theoretical saturation was reached and the final axial codes (category headings) identified. The GP and parent interviews were each separately concurrently analyzed alongside concurrent analysis of all the interviews, enabling the researcher to immerse herself in the individual GP and parent interview data as separate entities as well as the collective data. This added validity and rigor through demonstrating clarity and consistency in identification of the final axial codes.

## Results

Interviews were undertaken by the researcher. Data saturation was achieved after 29 parents and GPs interviewed: 11 parent interviews (9 mothers, 1 father, 1 joint mother and father) and 18 GPs (Table 2). Parent interviews took place in the home: 16 GP interviews in GP surgery, 2 in GP’s homes.

Parents of children aged 6-16 years with neuro-oncology (9) and solid (15) tumors participated. GP demographic data collected (Table 3) showed a range within each criterion and identified that nearly 25% of the GPs had been involved in more than one episode of children’s palliative care.

Five categories were identified during the collective analysis: GP role, Parent view of GP role, RCCC, Symptom management and Bereavement. Table 4 shows the final stage of categorizing the open codes into five axial codes. Due to the nature of the data collected, in some instances an open code was categorized within more than one axial code. For reader clarity the summary of the findings will be presented under the headings “GP experience” and “Bereaved parent experience.”

### GP experience

#### GP role

GPs saw their role as one where “you have to manage more or less anything that’s thrown at you” with specific roles identified of providing medication and supporting the family.

There was an acceptance that the child with cancer would disappear “… off the radar for quite a while …” (GP11) after diagnosis with the GP having little, if any, involvement during their active treatment. Although accepted GPs recognized that this contributed to difficulties some GPs faced re-engaging at the transition to palliation. Difficulties re-engaging were compounded by their relationship with the family being less well established than one they might have with an older patient entering palliation that they had “known for a long time” (GP7).

“I felt a bit awkward going in at this point … I was very conscious that they might have thought the GPs only interested in or daughter because she’s really poorly … maybe if I’d known her from diagnosis, kept regular contact, I think that would have made that transition into providing palliative care easier.” (GP14)

Time pressures were frequently cited as a factor influencing all aspects of GP practice: “With the pressures on time within general practice I think it’s getting increasingly hard to do the job as it should be done” (GP12). GPs recognized areas of practice that had changed as a result of time pressures. Where, for example, GPs might previously have regularly visited patients with cancer from diagnosis, time pressures now resulted in their visits being undertaken in response to a need rather than purely a supportive visit.

“… GPs don’t go and see the patients who are suffering from cancer once a week … they could go and see them and say hello … but because of the lack of time they can’t do it.” (GP5).

Family contact during palliation ranged from regular weekly GP visits to a single visit. GPs who had minimal family contact either took the stance, “There were people far more expert than me sort of doing it all and you know if I could add something then fantastic” (GP3), or likened it to their normal practice, “I never do routine visits in terminal care. I always leave it with them that I’m always there I’m on the phone they can call me at any time (GP 10). There was a perception of the need to get “… the balance between … not wanting to overload them with input but also not wanting them to feel unsupported …” (GP8).

#### RCCC

GPs acknowledged care provision would fall to them when the child returned home for palliation. The rarity of the experience, and GP’s concerns, made the offer of open contact with the RCCC welcome. The level and type of contact was not always clear, nor always alleviated the GP’s concerns at taking over managing the care.

“The secondary care consultant said … well my role has ended now pretty much and was there for advice …which is probably reasonable to be fair but it feels a little bit scary …” (GP4).

Joint GP and POONS home visits were acknowledged as opportunities to share knowledge and experience, but were rare in practice. Where they did occur it was recognized that “There’s a lot to learn from the people who are specialists” (GP21). For GP 19 listening to a conversation between a POONS and mother about how the child might die and what to do at the time of death, resulted in him reflecting on his practice and “not to shy away from talking about death when it’s inevitable.”

#### Symptom management

Effective symptom management is a key component of palliative care yet the rarity of child cancer deaths makes it difficult for GPs to gain or maintain knowledge. The uniqueness of the experience for GPs was evident in their concerns, “this is really scary we are not going to be able to do it” (GP8). Retrospectively the majority of GPs acknowledged that they had felt supported by the RCCC and gained confidence. Support was particularly welcomed when calculating doses for young children, the GPs were grateful they “… wouldn’t be left on (their) own trying to work out doses (for) small children” (GP8).

Although knowledge deficits were recognized, GPs saw themselves as “generalists and not specialists” (GP7) facing a rare event, and deemed addressing knowledge deficits “a futile gesture really” (GP4). Time pressures were an important factor, “you recognize … there’s something you don’t know … but don’t have time to find out much about it” (GP9), allocated training and education time was given to more commonly faced conditions. Involvement in the care itself was seen as a “good learning experience” (GP20) with GPs describing reflecting on, for example, the courage shown by a family (GP5).

#### Bereavement

The level of bereavement support provided was determined by the individual GP with none citing practice bereavement policies or guidelines. GP practice in bereavement ranged from, “I guess we expect people to ask for it … we wouldn’t offer anything routinely” (GP10) to those who routinely telephoned or sent cards to parents and invited parents to see them at the surgery. Once again time pressures were cited as a reason why visits were not undertaken and why contact was now more a “telephone call really just to pass on condolences” (GP20). Also identified was a concern of how their intervention might be construed by the family, whether they would they “be offering support or would we be intruding?” (GP2).

### Bereaved parent experience

#### Parent view of GP role

All the parents were glad they had been able to care for their child at home but for some families this had necessitated meeting new people at a difficult time. GPs fell into the category of “new” where families had limited previous contact (or in one case, had never previously met the GPs who visited them at home). Parents were adamant that they did not want to be meeting new professionals at this time, “You don’t want, at that end of time, to be having to be dealing with new people and new faces and neither did (child)” (Family (F) 8). Interactions were felt to lack “… any sort of … relationship with them because you didn’t know them” (F9). One parent recalled, “I didn’t want to talk to these strangers” (F24).

Regular home visits from the GP were seen to coincide with deterioration in the child’s health, “… he started coming really when she went downhill when she started getting really poorly at home and they said they didn’t give us much longer, you know a long time for her to live” (F4). For some families it was felt that a home visit earlier in the disease trajectory would have been beneficial, “… it would have been nice for (the GP) to have come to the family home before my son you know (before) his end of life (F21). Time pressures were recognized as being a factor influencing GP contact, “… would it have been nice … to just have … an occasional call or them to come and see how she was so that they knew the situation … But it’s time isn’t it … unfortunately everybody’s pushed to the enth degree and maybe that’s a luxury that you know as long as she’s being looked after then do we really need the GP to do that” (F17).

In the minority of cases GPs gave parents their personal telephone numbers in the terminal phase of the care, a gesture that was always welcomed by the family and not found to have been abused. F17 recalled their GP saying, “… call me at home any time of the night it doesn’t matter. I don’t care what time it is, if you’re worried about anything, call me and I always felt that I could have done.”

Parents predominantly saw their GP’s role in palliative care as one of providing prescriptions and also support, “making sure we were okay” (F4). Support was defined in terms of face-to-face contact with their GP.

#### RCCC

The transfer to primary care coinciding with the cessation of active treatment a difficult time for families, particularly when regular visits to the RCCC ceased. This was vividly portrayed by F4 “… you feel like your right arm is being cut off.” On-going open consultant contact was valued and appreciated; contact being made either through the POONS or by direct text/email. Maintaining links with the consultant was important to families, for some it was reassurance that the doctor who had managed their child’s care throughout active treatment and who knew their child’s medical background intimately was being kept informed. F8 appreciated being able to contact their consultant by text, stating that this was important because they “… didn’t want the medical care (of their son) to be sort of handed down … okay he’s dying now so I’m going to pass it down to a GP” (F8).

#### Symptom management

All families had access twenty-four hours a day, seven days a week to the POONS team for telephone advice but the level of involvement in symptom management from GPs varied. Parents recognized that GPs might not have the specialist knowledge, “… I didn’t feel that they had the knowledge really without being derogatory to help…” (F21) and where appreciative when this had been openly addressed “… I knew that my GPs had not been in that situation and that was absolutely fine because we were doing it together and they were open about it. Had they pretended that they knew and that was fine and I didn’t have to worry, it would have not reassured me” (F8).

#### Bereavement

Parents who saw their GP after the death reported their GP leaving, “an open door and said if you need to come and see me” (GP4). “Being bereaved” however was not always seen as a valid reason for parents to make an appointment to see their GP, “… you go if you’re unwell don’t you?” (F25). For some bereavement was perceived as something “they have to get on with” and they “gave an aura of being okay” (F17) when they saw people. Home visits were not expected by all the parents, “You have to approach them so I wouldn’t have expected them to come and do a home visit” (F25), but for others would have been a lifeline, “(A GP visit) would be good. I do find bereavement is a deep, dark place and once I think you do, there seems no purpose in life now, there’s nothing” (F25). Ensuring face-to-face contact after the death of a child might help identify those struggling or with abnormal grief.

## Discussion

Although it had been anticipated that each parent and their GP would be interviewed, due to the contact timings, some GPs were interviewed before parents declined participation. Determining sample numbers was informed through a grounded theory approach. GP recruitment, for example, ceased when a wide-range of GP characteristics were obtained in conjunction with theoretical saturation (identified through the chronological analysis of the GP interviews). Examining the family and their corresponding GP interview data provided an opportunity to quantify meaning behind actions or care, an example being Case 9, where the parent had commented “never the same GP came” and the GP detailed the way their practice had allocated and managed patients of a GP who recently retired, shedding light on why the family had seen different GPs during their child’s end of life care. Although the opportunity to examine the data in this way was not universal, the lack of universal dyad (both family and their GP interviewed) in all cases was not felt to influence findings.

All the GPs perceived they had a role to play in the care provision; the key GP roles identified by both the parents and the GPs themselves were those of providing support and medication.

The transition to palliation was difficult for both GPs and parents. Parents felt bereft when future hospital appointments were left “open” and GPs found re-engagement difficult. A lack of established relationship with their GP and poor understanding of their role resulted in parents perceiving that the management of their child’s care was being handed “down” from the “specialist,” the pediatric oncologist, to their GP. The majority of parents recalled their GP only becoming involved when their child became palliative and did not feel that palliation was a time to meet new people. Parents felt contact after diagnosis might have helped them develop a relationship with their GP and GPs themselves noted they lacked the familiarity with the family that they had with older palliative patients. Parents recognized that time pressures might prevent GPs from making contact. For the GP, loss of contact and difficulties re-engaging with the family [3,8] correlates with identified time pressures (GPs determining their best use of limited time), coupled with concerns over how/when to make contact and how the family may perceive their contact.

Time pressures were also felt to have influenced the perceived change in bereavement care; previously routine home visits being replaced by telephone calls or posted cards. Bereavement support was determined by individual GPs with none citing access to practice or national bereavement guidelines. Although bereavement support is seen as part of a GPs role [9,10], not all the parents viewed their GP as someone to see for bereavement support, highlighting a need for role clarity in bereavement. Interestingly, those that did have face-to-face contact with their GP felt they had been given “permission” to make future appointments. Although the majority of parents bereaved in this context do resolve their psychological morbidity over time [22] the journey is unique to each parent and any interventions offered need to be tailored to the individual. GPs need to differentiate those who would benefit from intervention from those who need none whilst balancing normalizing with not over-medicalising what, for many, will be a normal grief process in response to bereavement [9,23]. This is not an easy task as evidenced by bereaved parents struggling with their grief with no GP contact. A face-to-face meeting could enable GP’s to assess the grief response and may also facilitate (through ‘giving permission”) parents initiating future contact. Having awareness of bereavement support offered by different professionals, such as POONS and GPs, may result in more coordinated bereavement support being provided.

Knowledge deficits were identified by GPs but, despite availability of specialist on-line resources [24], it was felt more appropriate to target limited education time to more commonly seen conditions.

Patients dying at home are predominantly cared for by generalists such as GPs [25]. There is limited funding for formal education in, and limited availability of, pediatric focused post-graduate courses [15,26]. In this study, GPs recognized they could learn from being directly involved in the care and through undertaking joint visits with specialists. Through promoting collaborative working with specialists, such as POONS, GP learning can be optimized.

Findings support work [6] highlighting the role POONS can play in supporting the child, family and GP at this time. With their role transcending primary, secondary and tertiary settings POONS are ideally placed to facilitate on-going contact and reintegration of the GP into the care provision, reinforcing the GP’s role as part of the care team from the point of diagnosis, re-defining their role along the illness trajectory and facilitating joint home. Close collaboration and role clarity could help the GP achieve meaningful contact and address identified learning deficits within the time constraints they face. Clarity of the role of the GP in pediatric palliative care is integral to the future development of PPM, the GP being the one professional with potentially life-long involvement with the family.

Successfully gaining access to these participant groups and obtaining rich data from well-executed sensitive interviews, are key strengths of the study. Transferability is an important consideration for these findings as those who declined participation may have had different experiences. No parents of children under 6 years old or children with a leukemia diagnosis were recruited (the latter supporting data [27] showing more children with leukemia die in hospital). However findings can be considered of value to GPs working in children’s end of life care. Although children’s palliative care is thankfully a rare experience for GPs, findings from this study, such as enhancing collaborative working with specialists, are generalizable to other uncommon GP experiences.

## Conclusion

This study has highlighted the impact of time pressures on GP working practices. Findings also support prior research informing the provision of pediatric palliative care in the community. GPs find themselves with ever-increasing workloads and time pressures impact on their ability to both provide palliative and bereavement care and dedicate the necessary time to their education and learning in this rare but important field of practice. Findings have highlighted areas of practice where parents’ viewpoints could inform GP practice and enhance care provision. Enhanced communication and collaboration between pediatric oncology outreach nurses and GPs may help address identified challenges through promoting time-efficient role targeted working practices. Use of guidelines may also help GPs standardize best practice when they face this rare experience.

With the continued development of pediatric palliative medicine in the UK, accessibility to specialists in the field of children’s palliative care should increase. However findings suggest the GP will maintain their unique role in providing the continuity of care from diagnosis through bereavement support to on-going family medical care and will remain an important member of the inter-professional team providing palliative care to the child with cancer at home. It follows that GPs should have an active voice in the future development of pediatric palliative medicine.

## Figures and Tables

**Table 1:** Extract from interview topic guide.

Extract from GP topic guide	Extract from parent topic guide
i) What do you think the family saw as your role?	i) Can you tell me about your GPs role in providing the palliative care?
ii) What is your role in bereavement? (Are the parents registered on your caseload?)	ii) How do you feel about how the care was provided?
iii) Have you identified any educational needs since being involved in the care? What areas have you identified? How are these going to be addressed? Have you learnt from other health professionals involved in the care? What? Have you learnt from the family? What?	iii) Can you describe any difficulties or challenges you faced in providing the care?
	iv) What was good about the care X received?
	v) Is there anything else about X’s care that you would like to tell me about?

**Table 2:** Participant contact.

Case	Family Interview	GP Interview
**1**	Declined	Yes
**2**	Declined	Yes
**3**	Declined	Yes
**4**	Yes	Yes
**5**	Declined	Yes
**6**	Yes	Yes
**7**	No contact details	Yes
**8**	Yes	Yes
**9**	Yes	Yes
**10**	Yes	Yes
**11**	Declined	Yes
**12**	Yes	Yes
**13**	Yes	Yes
**14**	Declined	Yes
**15**	N/A (GP declined)	Declined
**16**	N/A	Declined
**17**	Yes	Yes
**18**	N/A	Declined
**19**	Declined	Yes
**20**	Declined	Yes
**21**	Yes	Yes
**22**	Declined	Not contacted
**23**	Yes	Not contacted
**24**	Yes	Not contacted

**Table 3:** GP demographic data extract.

Case	M/F	Age-range	Year qualified	Years as GP	Number of palliative children in career	Training
1	M	30-50	1990	8	2	8-9 years paediatrics
2	M	>50	1978	23	1	Surgical training
3	M	30-50	2003	3	1	Telephone (T) Face-to-face (F) Self-directed study (SDS)
4	M	31-50	1996	10	2	GP training
5	M	>51	1972	30	1	Course T / F consultant
6	M	>51	1973	28	1	T / F consultant SDS
7	M	>51	1978	27	1	T / F consultant
8	F	>51	1983	20	1	T / F consultant, Advice from POONS / community nurses
9	M	31-50	2001	3	1	Advice from POONS
10	F	31-50	1987	20	1	GP training
11	F	31-50	1987	20	1	T / F consultant SDS Learning from involvement
12	M	31-50	1992	14	2	T / F consultant SDS GP training Advice from POONS
13	M	31-50	1988	5	1	GP training Course
14	F	31-50	2004	2	1	T / F consultant SDS
17	M	31-50	1987	17	1	Not completed
19	M	31-50	1995	11	1	Not completed
20	F	31-50	1983	16	2	No training
21						Not completed

**Table 4:** Final stage of categorizing open codes (highlighted) into axial codes (bold).

**Axial code: GP role** Sources of GP information and learning (SII) View of care provision Communication Out of hours (OOH) Effect on GP GP support	**Axial code: Parent view of GP** Communication
**Axial code: Regional/shared care center** SII Communication OOH	**Axial code: Bereavement** View of care provision SII Communication
**Axial code: Symptom management** SII View of care provision GP learning GP information Communication OOH	
